# Comparison of Selected Kinematic Facebows Applied to Mandibular Tracing

**DOI:** 10.1155/2014/818694

**Published:** 2014-05-07

**Authors:** Mieszko Wieckiewicz, Marek Zietek, Danuta Nowakowska, Wlodzimierz Wieckiewicz

**Affiliations:** ^1^Division of Dental Materials, Faculty of Dentistry, Wroclaw Medical University, 50425 Wroclaw, Poland; ^2^Department of Periodontology, Faculty of Dentistry, Wroclaw Medical University, 50425 Wroclaw, Poland; ^3^Department of Prosthetic Dentistry, Faculty of Dentistry, Wroclaw Medical University, 50425 Wroclaw, Poland

## Abstract

The study focused on the comparison between mechanical and computerized registration methods used by the two selected kinematic facebows. The material consisted of 35 women aged 18 to 35, studied using the Gerber Dynamic Facebow and the computerized ARCUSdigma II axiograph. To compare the devices the condylar path inclination (CPI) was recorded according to the Camper's line, enabling the acquisition of easily comparable values based on which the devices were objectively and subjectively analyzed. Statistics was performed for the obtained data. The study showed that the values for the CPI registrated by the ARCUSdigma II are significantly higher than those obtained by using the Gerber Dynamic Facebow. The significant difference in the records of the CPI is most likely a result of the differences in the registration techniques assumptions. ARCUSdigma II provides the user with more diagnostic options than Gerber Dynamic Facebow. Mechanical facebow handling has a higher risk of hand-measuring errors in tracing procedure. Due to high discrepancy of achieved results from different systems the authors recommend to use articulator compatible with facebow whose measurement has been done.

## 1. Introduction

One of the prevailing problems of temporomandibular rehabilitation is the reliable evaluation of the dynamic relation between the occlusal surface and the condylar position [1–4]. One of the popular assessment methods is the use of facebows, allowing for three-dimensional diagnostics and enabling the upper jaw cast to be correctly placed in the articulator, as well as providing the user with the precise data necessary during the adjusting procedure [5–7]. This feature allows applying the facebows in fixed and removable dentures manufacturing. The kinematic facebows allow for individual dynamic evaluation of occlusion in relation to hinge axis, by geometrically assigning the distances between the occlusal plane and the rotation axis and then transferring the clinical data to the articulator [8, 9]. Therefore these devices are excellent tools allowing for functional rehabilitation of edentulous patients [10]. The kinematic systems provide precise and noninvasive diagnostics of temporomandibular joints movements and articular discs displacements [11]. Even though facebows are mechanical devices, also computerized variants are available [12–14].

The aim of the study was to compare the mechanical and computerized registration methods used by the two selected kinematic facebows.

## 2. Materials and Methods

**Figure 1:**
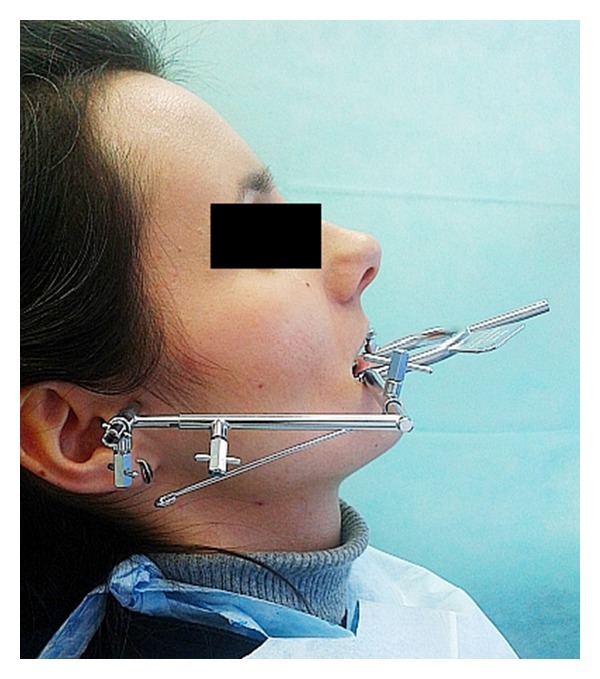
The Gerber Dynamic Facebow.

**Figure 2:**
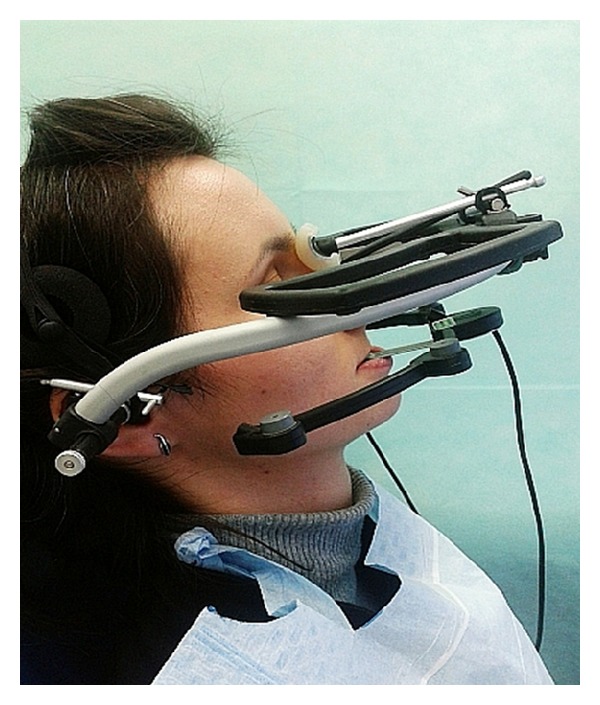
The computerized ARCUSdigma II axiograph.

The material consisted of 35 women who visited the Department of Prosthetic Dentistry of Wroclaw Medical University. Inclusion criteria were that the participant be aged between 18 and 35 years, be generally healthy female, be fully dentate, has not shown symptoms of the articular disc displacement, and has not revealed temporomandibular joint (TMJ) sounds, for example, crepitation or TMJ pain as well as limited mouth opening. Patients have been examined and qualified for the study in accordance with accepted standards contained on the official website of the International RDC/TMD Consortium [15]. The mean age in the studied groups was 25.71 (SD = 4.63). Patients were recorded using the Gerber Dynamic Facebow (Gerber Condylator, Zürich, Switzerland) and the computerized ARCUSdigma II axiograph (KaVo Dental Gmbh, Biberach, Germany) (Figures 1 and 2). The research was approved by the Bioethics Committee of the Wroclaw Medical University (decision number: KB-433/2012).

To compare the devices the condylar path inclination (CPI) was recorded according to the Camper's line, enabling the acquisition of easily comparable numerical data based on which the devices were objectively and subjectively analyzed.

The records for each patient were conducted for the left and right temporomandibular joint during protrusive movement. Hinge axis was aligned individually in each case. A reference position was standardized for each of the studied devices. The paraocclusal tray used by ARCUSdigma II was prepared each time by applying silicone on the occlusal surfaces and incisial margins of lower teeth. The reason for this was to increase the distances between teeth arches, obtaining results comparable with those recorded by using the upper registration plate with vertically adjustable pin and lower flat plate for the Gerber system. It should be emphasized that in each registration the authors tried to minimize the distance between teeth arches.

The Gerber Facebow was slid onto the lower plate which was in touch with the upper plate pin. The writing elements were opposite the marked reference position. A registration card was placed between the writing arm and the surface of the patient's TMJ area. The patients were asked to protrude their mandible. The writing element followed the condyle and recorded the path onto the card. Three tracings were made for each condyle. The angle of the CPI was determined for each chart with a protractor and then averaged. For each 1 mm of frontal bite opening between the incisors half a degree was added to the measured angle indicated on the registration cards according to the manufacturer's recommendation. Three condylar tracings were made also for ARCUSdigma II but after the last measurement device calculated the average angle automatically.

The results were analyzed using the* t*-test for two correlated samples, with statistical significance at  *P* ≤ 0.05. The software used in the statistical analysis was STATISTICA version 10 (StatSoft Inc., Tulsa, Oklahoma, USA).

## 3. Results

The average CPI recorded with ARCUSdigma II was 33.3° for the right side (SD = 10.58); 32.4° for the left side (SD = 13.93); total for both sides 32.9° (SD = 12.28).

The average CPI recorded with the Gerber Dynamic Facebow was 20.1° for the right side (SD = 9.94); 19.4° for the left side (SD = 9.40); 19.8° when measured as a whole (SD = 9.61). The CPI values measured during the study are presented in Table 1.

The study showed that the numerical results for the CPI recorded with the ARCUSdigma II are statistically significantly higher than those obtained by using the Gerber Dynamic Facebow. The results were then verified using the* t*-test for two correlated samples and the Kolmogorov-Smirnov test with Lilliefors' Significance. The results provided no reason against the initial hypothesis arguing that the distribution of statistical differences for each measured pair of data sets is statistically different from the average in each of the studied groups.

The* t*-test analysis of the CPI (right side) results indicates a statistical difference between the higher averages measured using the ARCUSdigma II [*t*(df : 34) = 7.18;  *P* < 0.00001] and the lower averages obtained using the Gerber Dynamic Facebow.

The* t*-test analysis of the CPI (left side) results indicates a statistical difference between the higher averages measured using the ARCUSdigma II [*t*(df : 34) = 7.18;  *P* < 0.00001] and the lower averages obtained using the Gerber Dynamic Facebow.

The* t*-test analysis of the CPI (combined results, left and right side) results indicates a statistical difference between the higher averages measured using the ARCUSdigma II system [*t*(df : 69) = 10.18;  *P* < 0.00001] and the lower averages obtained using the Gerber Dynamic Facebow.

## 4. Discussion

According to the literature and the authors' clinical experience, it can be argued that women constitute a higher risk in the development of temporomandibular disorders [16–19]. For this reason the study included only female patients, that is, patients more often affected with the abovementioned problem. The age range was chosen due to the lower risk of pathological changes in condylar morphology among young people [20]. Examined group characteristics allowed achieving an unequivocal and reliable outcome.

The experiment showed that the values obtained for the condylar path inclination vary significantly depending on the device used. This result is surprising, as each individual evaluation using both facebows was always conducted using the same patient and the same researcher operating the devices. The CPI measured using the computerized ARCUSdigma II axiograph was significantly higher than the results recorded by Gerber Dynamic Facebow. The difference is most likely a result of the registration techniques varieties.

The study using the Gerber Dynamic Facebow is based on the mechanical sagittal registration of the condylar movement and the manually calculating values for the condylar path inclination. The condylar path tracing is measured according to the occlusal plane which is almost parallel to Camper's line. Therefore the measurement can also refer to this line objectively. The registration also provides information about the length and shape of condylar path. The starting point for the stylus arm is located in the arc's facial manually. It should be noted that the condylar process is not a point and the researcher should be aware of its complex shape. Gerber Facebow was selected for testing because of its popularity and useful clinical features.

The ARCUSdigma II facebow is a computerized axiograph. Its operation is based on the analysis and imaging of the hinge axis and its movement, used to calculate the necessary parameters, that is, condylar path inclination, Bennett's angle, immediate side shift, and Bennett's shift, allowing for a qualitative on-screen computer analysis of mandibular movements. The recording is preceded by determining of the reference position, which is set by the device by establishing the arrangement of ultrasonic emitters and microphones. The entire test is analyzed by the device's software.

The difference in the recording techniques may lead to a statistically significant change in the results for the CPI, which is an important factor in the rehabilitation of the masticatory system. Hernandez et al. [21] in their studies present the mean values for the condylar inclination (right and left side) of the TMJ, in test group studies by Cadiax system, which were higher than the values recorded in the research. The study conducted by Kucukkeles et al. [22] showed that the measurements carried out by means of mechanical and electronic axiography did not differ significantly. These small differences may be due to inaccuracy of the manual technique. Petrie et al. [23] reported a result similar to the result obtained in the experiment conducted by the authors. By comparing the mandibular tracing recorded by computerized axiograph and mechanical pantograph they identified discrepancies in the values. This result confirms that the registrated condylar path inclination may vary depending on recording techniques. It should be noted that no comparative studies using kinematic facebows identical to those used in followed research have been found in the available literature.

According to the authors the Gerber Facebow is a device easier to use than the ARCUSdigma II. However, the Gerber system offers fewer diagnostic capabilities. The time required to perform the measurement, including the setting of the facebow on the patient's head, speaks strongly in favor of the mechanical device. The Gerber system does not depend on the proper functioning of the computer or power supply. It should be noted that extensive menu in the electronic device and the visualization of the mandibular movements on a desktop allows full three-dimensional diagnostics of the temporomandibular joints moves, which is the great clinical value. The ARCUSdigma II function of storing recorded data on a hard drive or SD card is of great convenience in comparison with the paper recording system required by the Gerber facebow. With just a single measurement the electronic device allows the user to obtain more data, being also able to adjust the individual or semi-individual articulator. Using the standard facebow does not provide the specialist with such choice. The smaller diagnostic potential of the Gerber platform, however, does not make it an obsolete tool in clinical application.

It should be noted that virtual tools for mandibular tracing with extremely high diagnostic potential will be introduced to the daily practice in close future [24–26].

## 5. Conclusions

1. The significant difference in the measurements of the condylar path inclination is most likely a result of the differences in the registration techniques assumptions.
2. ARCUSdigma II provides the user with more diagnostic options than Gerber Dynamic Facebow.
3. Mechanical facebow handling has a higher risk of hand-measuring errors in tracing procedure.
4. Due to high discrepancy of achieved results from different systems the authors recommend to use articulator compatible with facebow whose measurement has been done.

## Figures and Tables

**Table 1:** Condylar path inclination values recorded by the ARCUSdigma II and Gerber Dynamic Facebow.

No.	ARCUSdigma II		Gerber Dynamic Facebow	
	Right TMJ	Left TMJ	Right TMJ	Left TMJ
1	43.2	47	15.4	20.4
2	26.8	21.2	22.5	23
3	36.4	11.2	34	10
4	17.8	22.2	14.5	20
5	26.2	13.8	24	16
6	13.8	8.2	12.5	10
7	36.7	34.8	19	27
8	12.1	4.8	12	8.7
9	21	47.2	20	41
10	39.7	54.5	33	30
11	12.1	25.7	14.3	20.7
12	20.7	30	15.3	19.7
13	19.7	20	4.3	23.7
14	26.1	12.3	13.7	11
15	42.1	27.1	22.4	20
16	38.6	44.9	11	13
17	42.5	43.2	41.7	40.7
18	45	44.2	29.3	24
19	45	45	25.7	27.3
20	34.8	31.1	7.5	3.3
21	32.7	38.3	23.7	21
22	32.7	30.4	40.7	15.3
23	53.6	53.3	25.3	22.7
24	36.4	42.8	30	19.7
25	43.7	36.9	26	12.3
26	24	28.5	5	7
27	41.6	45	26	28.7
28	31.8	21.4	6.7	6.7
29	35	35	25.3	19.7
30	37.1	45	28.3	28.3
31	31.6	40.2	12	28.7
32	34.8	23.3	5.7	6.3
33	45.1	12	10.7	8
34	42.1	49.8	30	31.7
35	42.8	45	14.7	15
Average (SD)	**33.3 (10.58)**	**32.4 (13.93)**	**20.1 (9.94)**	**19.4 (9.4)**

## References

[1] Noh K, Choi W, Pae A, Kwon KR (2013). Prosthetic rehabilitation of a patient with unilateral dislocated condyle fracture after treatment with a mandibular repositioning splint: a clinical report. *The Journal of Prosthetic Dentistry*.

[2] Endo M, Terajima M, Goto TK, Tokumori K, Takahashi I (2011). Three-dimensional analysis of the temporomandibular joint and fossa-condyle relationship. *Orthodontics: The Art and Practice of Dentofacial Enhancement*.

[3] Weffort SY, de Fantini SM (2010). Condylar displacement between centric relation and maximum intercuspation in symptomatic and asymptomatic individuals. *The Angle Orthodontist*.

[4] Okano N, Baba K, Akishige S, Ohyama T (2002). The influence of altered occlusal guidance on condylar displacement. *Journal of Oral Rehabilitation*.

[5] Small BW (2009). Recording jaw relationship records: facebow, stick bites, or both?. *General dentistry*.

[6] O’Malley AM, Milosevic A (2000). Comparison of three facebow/semi-adjustable articulator systems for planning orthognathic surgery. *British Journal of Oral and Maxillofacial Surgery*.

[7] Bowley JF, Michaels GC, Lai TW, Lin PP (1992). Reliability of a facebow transfer procedure. *The Journal of Prosthetic Dentistry*.

[8] Bakalczuk M, Bozyk A, Iwanek M, Borowicz J, Sykut J, Kleinrok J (2004). Diagnostic abilities of three-dimensional electronic axiography on the basis of ARCUSdigma device. *Annales Universitatis Mariae Curie-Sklodowska D: Medicina*.

[9] Stiesch-Scholz M, Demling A, Rossbach A (2006). Reproducibility of jaw movements in patients with craniomandibular disorders. *Journal of Oral Rehabilitation*.

[10] Dubojska AM, White GE, Pasiek S (1998). The importance of occlusal balance in the control of complete dentures. *Quintessence International*.

[11] Ahangari AH, Torabi K, Pour SR, Ghodsi S (2012). Reproducibility of jaw movements in patients with craniomandibular disorders. Evaluation of the Cadiax Compact II accuracy in recording preadjusted condylar inclinations on fully adjustable articulator. *The Journal of Contemporary Dental Practice*.

[12] Samet N, Smidt A, Samet N, Weiss EI (2002). A clinical and cost-benefit evaluation of five facebows. *Quintessence International*.

[13] Franklin P, McLelland R, Brunton P (2010). An investigation of the ability of computerized axiography to reproduce occlusal contacts. *The European Journal of Prosthodontics and Restorative Dentistry*.

[14] Krzemień J, Baron S (2013). Axiographic and clinical assessment of temporomandibular joint function in patients with partial edentulism. *Acta of Bioengineering and Biomechanics*.

[15] Ohrbach R, Gonzalez Y, List T, Michelotti A, Schiffman E Diagnostic Criteria for Temporomandibular Disorders (DC/TMD) Clinical Examination Protocol.

[16] Köhler AA, Hugoson A, Magnusson T (2012). Prevalence of symptoms indicative of temporomandibular disorders in adults: cross-sectional epidemiological investigations covering two decades. *Acta Odontologica Scandinavica*.

[17] Liu F, Steinkeler A (2013). Epidemiology, diagnosis, and treatment of temporomandibular disorders. *Dental Clinics of North America*.

[18] di Paolo C, Costanzo GD, Panti F (2013). Epidemiological analysis on 2375 patients with TMJ disorders: basic statistical aspects. *Annali di Stomatologia*.

[19] Köhler AA, Hugoson A, Magnusson T (2013). Clinical signs indicative of temporomandibular disorders in adults: time trends and associated factors. *Swedish Dental Journal*.

[20] Mathew AL, Sholapurkar AA, Pai KM (2011). Condylar changes and its association with age, TMD, and dentition status: a cross-sectional study. *International Journal of Dentistry*.

[21] Hernandez AI, Jasinevicius TR, Kaleinikova Z, Sadan A (2010). Symmetry of horizontal and sagittal condylar path angles: an *in vivo* study. *The Journal of Craniomandibular & Sleep Practice*.

[22] Kucukkeles N, Ozkan H, Ari-Demirkaya A, Cilingirturk AM (2005). Compatibility of mechanical and computerized axiographs: a pilot study. *The Journal of Prosthetic Dentistry*.

[23] Petrie CS, Woolsey GD, Williams K (2003). Comparison of recordings obtained with computerized axiography and mechanical pantography at 2 time intervals. *Journal of Prosthodontics*.

[24] Gąska D, Kijak E, Lipski T, Margielewicz J (2012). Numerical modelling of human masticatory organ kinematics. *Mechanika*.

[25] Keeling AJ, Brunton PA, Holt RJ (2014). An *in vitro* study into the accuracy of a novel method for recording the mandibular transverse horizontal axis. *Journal of Dentistry*.

[26] Solaberrieta E, Mínguez R, Barrenetxea L, Etxaniz O (2013). Direct transfer of the position of digitized casts to a virtual articulator. *The Journal of Prosthetic Dentistry*.

